# Reliability of CBCT in the diagnosis of dental asymmetry

**DOI:** 10.1590/2176-9451.19.2.090-095.oar

**Published:** 2014

**Authors:** Antônio Carlos de Oliveira Ruellas, Leonardo Koerich, Carolina Baratieri, Claudia Trindade Mattos, Matheus Alves Junior, Daniel Brunetto, Lindsey Eidson

**Affiliations:** 1 Phd in Dentistry and Adjunct Professor, Federal University of Rio de Janeiro (UFRJ); 2 MSc in Orthodontics, UFRJ; 3 Phd in Orthodontics, UFRJ; 4 Phd in Orthodontics, UFRJ. Adjunct Professor, UFF; 5 Doctorate student in Orthodontics, UFRJ; 6 MSc in Orthodontics, University of North Carolina

**Keywords:** Cone-beam computed tomography, Imaging, Three-dimensional diagnosis, Dental arch

## Abstract

**Objective:**

The aim of this study was to validate a method used to assess dental asymmetry, in relation to the skeletal midline, by means of CBCT.

**Methods:**

Ten patients who had CBCT scans taken were randomly selected for this study. Five different observers repeated 10 landmarks (x, y and z variables for each) and 12 linear measurements within 10 days. Measurements were taken in both arches to evaluate symmetry of first molars, canines and dental midline in relation to the skeletal midline. Intraclass correlation coefficient (ICC) was carried out to assess intra- and interobserver reliability for landmarks and distances. Average mean difference was also assessed to check measurement errors between observers.

**Results:**

ICC landmarks was ≥ 0.9 for 27 (90%) and 25 (83%) variables for intra- and interobserver, respectively. ICC for distances was ≥ 0.9 for 7 (58%) and 5 (42%), respectively. All ICC landmarks for distances were >0.75 for both intra- and interobserver. The mean difference between observers was ≤ 0.6 mm for all the distances.

**Conclusion:**

The method used to assess dental asymmetry by means of CBCT is valid. Measurements of molars, canines and dental midline symmetry with the skeletal midline are reproducible and reliable when taken by means of CBCT and by different operators.

## Abstract (pt)

**Objetivo:**

validar um método para avaliar assimetria dentária, em relação à linha média esquelética, usando TCFC.

**Métodos:**

dez pacientes que realizaram TCFC foram selecionados aleatoriamente para esse estudo. Cinco diferentes observadores repetiram 10 pontos de referência (com variáveis x, y e z, para cada ponto) e 12 medidas lineares em um intervalo de 10 dias. As medições foram realizadas em ambas as arcadas, para avaliar a simetria de primeiros molares, caninos e linha média dentária, em relação à linha média esquelética. Índice de correlação intraclasse (ICC) foi realizado para verificar a confiabilidade intraobservador e interobservadores para os pontos de referência e distâncias. A diferença média também foi avaliada, para checar os erros de mensuração entre os observadores.

**Resultados:**

ICC para os pontos de referência foram ≥ 0,9 para 27 (90%) e 25 (83%) das variáveis para intra- e interobservadores respectivamente. ICC para distâncias foi ≥ 0,9 para 7 (58%) e 5 (42%), respectivamente. Todas as medidas de ICC para distâncias foram > 0,75 para intraobservador e interobservadores. A diferença média entre observadores foi ≤ 0,6mm para todas as distâncias.

**Conclusão:**

o método de verificação de assimetria dentária utilizando TCFC é válido. Medições de molares, caninos e linha média dentária com a linha média esquelética são reproduzíveis e confiáveis quando feitas utilizando TCFC, mesmo que por diferentes operadores.

## INTRODUCTION

Patients with malocclusion often present one or more characteristics related to asymmetry, for instance, Class II or III subdivision, dental midlines that are not coincident with each other, and/or dental midlines that are not coincident with the facial midline.^1^ Proper orthodontic treatment planning requires a correct diagnosis. Dental arch rotation on the vertical axis, known as yaw, is often omitted in classifications and diagnosis. This important piece of information can determine the need for asymmetric mechanics or extractions to correct a dental midline shift or a unilateral Class II or III relationship, for example.^2^

Different methods can be used for diagnosis of patient's dental symmetry in relation to the skeletal midline (midsagittal plane). Burstone^1^ has suggested, within a few limitations, the use of posteroanterior radiography to evaluate maxillary and mandibular discrepancies and the upper and lower dental midlines in relation to the skeletal midline. Another method suggests that the median raphe is the patient's skeletal midline.^3^ In this method, the relationship between teeth and bone can be analyzed by means of dental casts. Furthermore, the methods described by Moyer^3^ or Proffit^4^ can help to identify asymmetry by means of a ruler and a bow divider or a symmetric grid, respectively. More recently, advances in technology have allowed the transfer of plaster models to a computer by using scanners.^5^ They have also enabled three-dimensional models to be created on the basis of data obtained from Cone Beam Computed Tomography (CBCT), reproducing the patient's teeth and surrounding bone structures.^6^ These models, however, are not linked to the patient's face anatomy; therefore, the advantages that a CBCT can provide, such as skeletal and dental diagnosis, are not used to their full potential. With a view to addressing such issue, some computer programs allow navigation in CBCT data through tomographic slices taken in the three planes of space and, with adjustment of the threshold, it is possible to visualize, at the same time, the teeth, bone and soft tissues.^7^ Thus, the aim of this study was to validate a method used to evaluate, by means of CBCT, dental asymmetry (molars, canine and dental midline) in relation to the skeletal midline.

## MATERIAL AND METHODS

Sample size calculation was carried out (α = 0.05; β = 0.2; ρ_0_ = 0.45; ρ_1_ = 0.90)^8^ and revealed that ten patients would be enough for 10 observations (twice, 5 observers). This study, approved by the Federal University of Rio de Janeiro Institutional Review Board, comprised ten patients who were being orthodontically treated and had CBCT taken. Patients were randomly selected. In selecting the sample, the following exclusion criteria were applied: absence of canines and incisors; presence of restorations at the evaluated sites; and syndromes, such as cleft lip and palate, by which maxillary bone formation could be affected.

The CBCT equipment used was an i-CAT (Imaging Sciences, Hatfield, PA), with a 13 x 17 cm field of view, voxel dimension of 0.4 mm and exposure time of 20 seconds. The images were obtained at 120 kVp and 5 mA. All patients were in maximum intercuspation during the scan.

**Figure 1:**
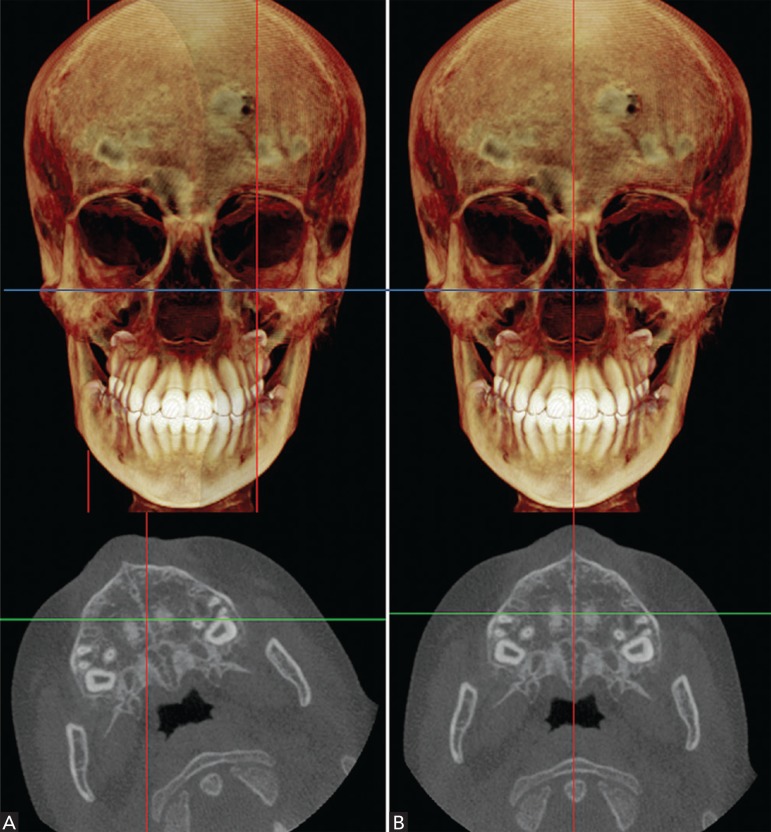
**A**) Example of a patient with the Frankfort Horizontal Plane horizontally oriented. **B**) After one operator reoriented the skeletal midline with the sagittal plane (red).

After the images were taken, one operator imported all DICOM (Digital Images and Communication in Medicine) files into Dolphin 3D (Dolphin Imaging, version 11.0, Chatsworth, CA) software. For standardization purposes, the Frankfort Horizontal Plane was horizontally oriented for all patients. In addition, slice thickness was set to be equal to the voxel size. Patients' data were saved and all the observers started taking the measurements at this point. Each observer had to orient the patient's head (turning to left or right, only) and had to try to match the skeletal midline with the sagittal plane (Fig 1), using nasion, anterior nasal spine and posterior nasal spine as reference, before beginning the analyses.

**Figure 2:**
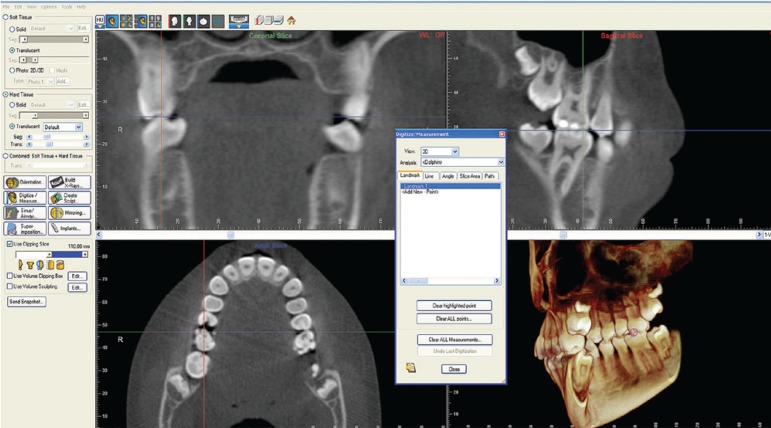
Example of landmark positioning. After being identified in three different slices, the landmark was plotted in the axial view of the multiplanar reconstruction (lower left box).

**Figure 3:**
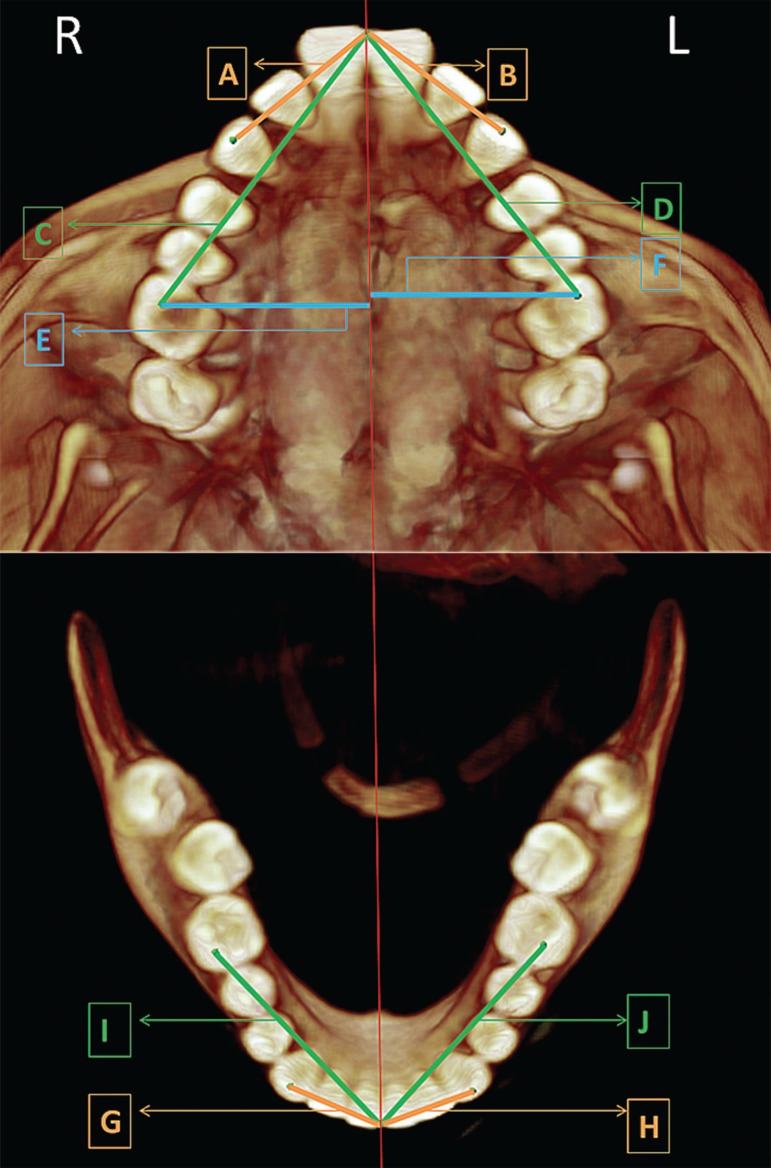
Linear distances as shown in Table 2.

**Figure 4:**
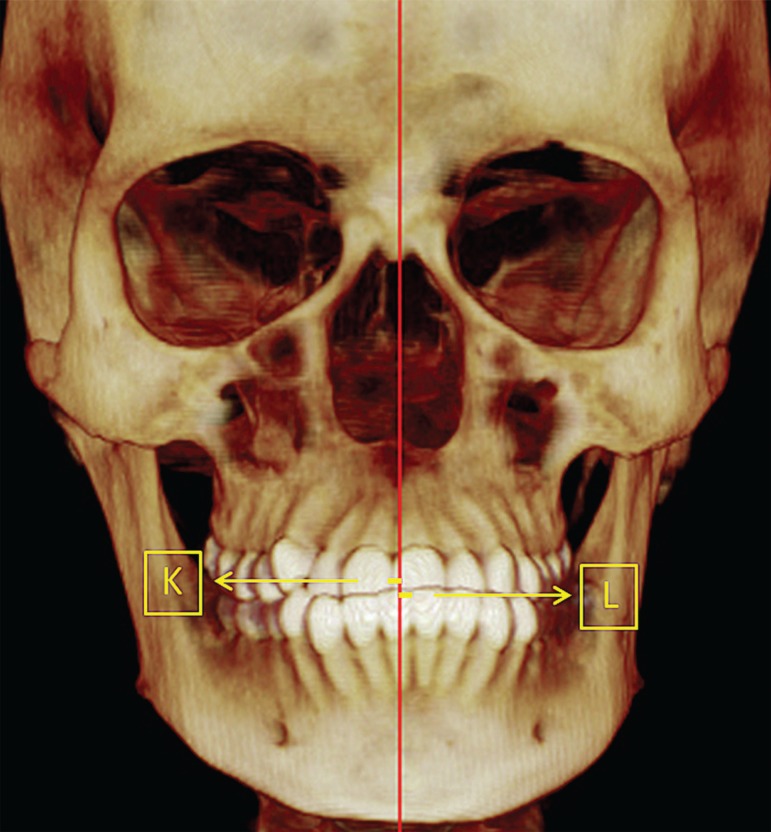
Linear distances as shown in Table 2.

Five different observers - all students of Orthodontics, with one to two years of experience working with CBCT - were asked to test the reproducibility of 10 landmarks and 12 distances using the CBCT scans, as shown in Tables 1 and 2. Calibration was done with two scans that were not included in the sample. Evaluations were carried out independently and repeated within an interval of ten days. For more accuracy in the following step, the size of the landmarks was set at 0.01 mm. All four views (sagittal, axial, coronal and the rendered image) were used as reference to locate the landmarks. However, landmarks were only plotted in the axial slices of the multiplanar reconstruction (Fig 2). Figure 3 and 4 show the distances between the landmarks used in the study.

**Table 1:** Localization of the landmarks used in the study.

Landmark	Anatomic region	Coronal slice	Axial slice	Sagittal slice
**Maxilla**				
UR6	Right molar mesiobuccal cusp tip	Middle-inferior-most point	Middle point	Middle-inferior-most point
UR3	Right canine cusp tip	Middle-inferior-most point	Middle point	Middle-inferior-most point
UML	Skeletal midline at upper incisors incisal edge	Middle-inferior-most point between incisors	Middle point between incisors	Anterior-inferior-most point
UL6	Left molar mesiobuccal cusp tip	Middle-inferior-most point	Middle point	Middle-inferior-most point
UL3	Left canine cusp tip	Middle-inferior-most point	Middle point	Middle-inferior-most point
**Mandible**				
LR6	Right molar mesiobuccal cusp tip	Middle-superior-most point	Middle point	Middle-superior-most point
LR3	Right canine cusp tip	Middle-superior-most point	Middle point	Middle-superior-most point
LML	Skeletal midline at lower incisors incisal edge	Middle-superior-most point between incisors	Middle point between incisors	Anterior-superior-most point
LL6	Left molar mesiobuccal cusp tip	Middle-superior-most point	Middle point	Middle-superior-most point
LL3	Left canine cusp tip	Middle-superior-most point	Middle point	Middle-superior-most point

**Table 2:** Distance between landmarks.

Maxilla	
Distance A	Distance between UR3 and UML
Distance B	Distance between UL3 and UML
Distance C	Distance between UR6 and UML
Distance D	Distance between UL6 and UML
Distance E	Distance between UR6 90º to the skeletal midline
Distance F	Distance between UL6 90º to the skeletal midline
**Mandible**	
Distance G	Distance between LR3 and LML
Distance H	Distance between LL3 and LML
Distance I	Distance between LR6 and LML
Distance J	Distance between LL6 and LML
**Midline**	
Distance K	Distance between the skeletal midline and the midline of the upper teeth
Distance L	Distance between the skeletal midline and the midline of the lower teeth

Landmarks and distances were obtained by means of the Digitize/Measurement tool available in the 3D view of the software. After all landmarks were plotted, the next step was to measure the distance between them. The software did not allow automatic connection between two landmarks. For this reason, this step had to be taken manually. To calculate the distance between two landmarks, the observer only connected the landmarks of interest. Both landmarks and distances were exported to Microsoft Excel (Microsoft Corporation, Redmond, WA).

## STATISTICAL ANALYSES

Analyses were carried out with the Statistical Package for the Social Sciences 17.0 (Chicago, IL, USA). Intra-examiner and inter-examiner reliability values for both landmarks and distances were determined by using intraclass correlation coefficients (ICCs). Average mean differences for the distances measured by different examiners (measurement errors) were summarized, and descriptive statistics were applied. The paired t-test was also applied to detect significant mean differences. The level of significance was set at 0.05.

## RESULTS

The reliability in defining the landmarks was estimated by ICC for each coordinate of each landmark. As a result, 30 variables (x, y and z for each landmark) were tested. The ICC was ≥ 0.9 for 27 (90%) of all intraobserver assessments, and the lowest intraobserver coefficient was 0.706. The ICC was ≥ 0.9 for 25 (83%) for all interobserver assessments, and the lowest interobserver coefficient was 0.591.

Table 3 shows the frequency of intraobserver and interobserver reliability estimated by ICC for the distances measured.

**Table 3:** Frequency of intra and interobserver reliability estimated by intraclass correlation coefficient (ICC) for the distances measured.

Values	Intraobserver		Interobserver	
	n	(%)	n	(%)
ICC ≥ 0.90	7	58	5	42
0.75 < ICC < 0.90	5	42	4	33
0.45 < ICC ≤ 0.75	0	0	3	25
ICC ≤ 0.45	0	0	0	0
Total	12	100	12	100

Table 4 shows the frequency of the mean difference for the distances measured by each observer. The mean difference was calculated using paired t-tests performed between every two observers for each distance. The results are summarized in Table 4 and illustrate that 10 (83%) measurements had a very small mean difference of less than 0.5 mm and no measurement had a mean difference greater than 1 mm.

**Table 4:** Frequency of the mean difference among observers on the distances measured.

Values (mm)	n	(%)
≥ 2	0	0
1 < x < 2	0	0
0.5 < x ≤ 1	2	17
≤ 0.5	10	83
Total	12	100

Table 5 lists the reliability estimated by ICC and the interobserver mean difference for each distance.

**Table 5:** Reliability estimated by intraclass correlation coefficient (ICC) for each distance.

Distances	Intraobserver reliability	Interobserver reliability	Interobserver mean difference (mm)
A	0.932	0.920	0.31
B	0.883	0.859	0.34
C	0.959	0.934	0.35
D	0.969	0.900	0.54
E	0.886	0.916	0.60
F	0.949	0.867	0.41
G	0.813	0.862	0.23
H	0.917	0.741	0.26
I	0.893	0.866	0.50
J	0.963	0.946	0.22
K	0.781	0.591	0.35
L	0.958	0.740	0.38

## DISCUSSION

Only skeletal structures were used to define the skeletal midline in this study. The references used were landmarks such as anterior and posterior nasal spine and nasion. Differently from other studies using CBCT,^9,10^ only the Frankfort Horizontal Plane was pre-oriented and each individual observer later established the skeletal midline. The reason was that if the head was already oriented with the skeletal midline in the sagittal plane, it would increase the likelihood for bias and make it easier for each observer to define the plane. Head orientation does not influence linear measurements;^11^ as long as the same landmarks were obtained, measurements should be the same.

Grauer et al^7^ demonstrated that landmarks are better located when plotted in the stack of slices rather than in rendered images. This technique was employed by our study of which results corroborate the findings of other researches that showed high values for intraclass^9,10^ and interclass^9,12^ correlation for landmarks identified in dental structures.

Creed et al^6^ showed that anteroposterior measurements for molars can be reliably taken using either digital models or surface models made on the basis of CBCT data. Asquith et al^13^ investigated dental casts and 3D digital study models and found that intraexaminer mean differences for this variable were ≤0.05 mm and ≤0.32 mm, respectively. Our study had slightly higher mean differences; however, it was interexaminer instead of intraexaminer. In addition, the values were not clinically significant (all of them ≤ 0.54 mm). The present research also confirmed that the same type of anteroposterior evaluation can be applied for the canines.

Mean difference between observers for distances from skeletal to dental midlines were ≤ 0.4 mm. The other transversal measurement, molars perpendicular line to the skeletal midline, showed good reliability between observers. Other techniques have been applied for this evaluation. However, conventional or 3D digital models can use only the palatal rugae as reference, which is reliable for growing patients.^14^ Nonetheless, using the raphe as the skeletal midline may not be the best option, as it has different shapes and curvatures.^1^ Nevertheless, skeletal midline and raphe have been associated in the past.^15^ With 3D surface models, one can obtain other structures that would likely provide a reliable skeletal midline. However, the production of these models involves either hiring a specialized company, which implies in higher costs,^6^ or computer expertise, which is extremely time consuming.^9,16^ To our view, the process involved in any of these options does not outweigh the benefits.

The advantages of the proposed method are as follows: the possibility of assessing and reproducing patients' skeletal midline and relating it to the teeth and soft tissues, and the possibility of directly taking measurements in the CBCT slices by means of simple techniques. Based on recent controversies, the main disadvantage is that not every patient needs a CBCT scan. Additionally, even though it is an important piece of data that can be obtained for cases of skeletal asymmetry, we do not recommend that CBCT scans be taken for this purpose only. In spite of being recommended for very specific cases, CBCT scans have lower radiation doses,^17,18^ lower costs and good accuracy.^19^ For this reason, the exam has been increasingly used, in addition to becoming more accepted.^20^ The radiation doses involved in this type of exam are similar to those of a full-mouth series of radiographs. Furthermore, one single CBCT scan is able to provide data for airway, sinus and TMJ analyses.^21,22^ On the other hand, another drawback is the potential presence of artifacts in the areas of interest and the need for specific software for evaluation.

Clinically determining dental midline shifts using the soft tissue as reference can be misleading when there are asymmetries in nose, chin or philtrum.^23^ The proposed "imaginary plumb" method^24^ as a true vertical line is affected by the patient and operator position as well as the parallax effect.

Anteroposterior dental asymmetry is often present in subdivision malocclusions. It can be corrected by means of minor dental movements or extractions depending on the degree of the discrepancy. It is necessary to diagnose in which arch and side the asymmetry is located to decide which mechanics will be applied. The evaluation on dental casts will use the raphe as the skeletal midline, but some degree of variation might occur between different operators due to the shape of the raphe. Therefore, evaluating dental asymmetry by means of CBCT images and having the skeletal midline as reference provides useful information for diagnosis.

## CONCLUSION

Measurements for molars, canines and incisors in relation to the skeletal midline taken to assess dental asymmetry are reproducible and reliable when taken by means of CBCT.
